# Burnout among Healthcare Professionals in Ghana: A Critical Assessment

**DOI:** 10.1155/2020/1614968

**Published:** 2020-03-21

**Authors:** Stephen T. Odonkor, Kwasi Frimpong

**Affiliations:** School of Public Services and Governance, Ghana Institute of Management and Public Administration, Accra, Ghana

## Abstract

Health workers are prone to burnout, which can have an adverse effect on their person and the patients to whom care is offered. The goal of this paper was to assess the levels of burnout experienced by healthcare workers in Accra, Ghana. The study was conducted using the cross-sectional study design. Questionnaires were used to obtain data from 365 respondents who worked in 12 major healthcare facilities. Data obtained were analyzed with SPSS version 23. Majority of the respondents were females (56.7%) as against males (43.3%). The total score for all burnout variables among health worker groups ranged from good (71.50%), alarming (12.60%), acute crisis (6.02%), and burnout (9.90%). Among the health worker groups, nurses had the highest percentage score values for all burnout variables. There was an association between burnout and these sociodemographic characteristics: age (*p* < 0.001), gender (*p* = 0.003), educational qualification (*p* < 0.001), occupation (*p* < 0.001), years of work experience (*p* < 0.001), marital status (*p* < 0.001), and parenthood (having children) (*p* < 0.001). It is recommended that measures should be put in place in Ghanaian hospitals to assess stress and burnout levels to ensure people who are going through such situations are properly cared and supported.

## 1. Introduction

Burnout poses health risk among working populations especially young people, yet it has largely been neglected as a result of the increasing work pace, coupled with the rapidly growing demands on workers [1]. Burnout is defined as “complete emotional, physical, and mental exhaustion” [2]. A burnt-out person experiences further frustration because of inability to perform on the job. Burnout presents a challenge that transcends all occupations and professions especially for care- and service-based professions such as health workers. Employees experiencing elevated levels of burnout, although seldom complain to colleagues or supervisors, tend to demonstrate apathy to their job roles and schedules. Absenteeism and employee turnover are key manifestations of burnout. Furthermore, burnout is also known to negatively affect productivity, lower job satisfaction, and decrease organizational citizenship behaviors.

It is estimated that healthcare workforce represents 12% of the working population worldwide [3]. Healthcare professionals work in groups (multidisciplinary specialized team of experts) that support and assist the health and well-being of mankind. This places a high demand on their team members; thus, they face the risk of burnout. This is further compounded because healthcare professionals work in an environment that is cogitated to be one of the most hazardous occupational settings [4, 5]. Indeed, it is worth noting that the attention to burnout was first brought to light as a result of the situation of nurses in hospice care [2].

Transience of life, helplessness, sufferings, futile battle, and grief are encountered by the healthcare worker on a daily basis. Thus, it is of importance to take care of their psychological well-being, which may subsequently influence the well-being of the patients who have been entrusted under their care and supervision [6]. Furthermore, burnout and low engagement in healthcare setting may negatively affect patient care, undermine the workforce, and rise turnover [7]. This presents with interruption in continuity of care and associated high cost with regard to hiring new healthcare professionals. Invariably, it is believed that where there is a happy caregiver, there is also a satisfied patient [8]; thus, an output of high-quality care for clients and patients alike must necessarily be preceded by high-quality care for the health worker.

In sub-Saharan Africa, not much attention has been given to burnout issues among health workers, as most attention is directed mainly towards the occupational health and safety of the health worker due to diverse hazards related to their work-related activities [7, 9–11]. However, a burnout worker is much more prone to occupational hazards. Secondly, in situations where burnout has been investigated, it is usually limited to just a few categories of health workers; thus, one cannot determine the trends among the diverse category of health workers. Additionally, the growing attention to burnout and employee engagement in healthcare must be matched by better evidence about how burnout affects the workforce, patient care, and healthcare organizations [7, 12, 13]. The goal of this paper was to assess the levels of burnout experienced by healthcare workers, aimed at identifying the various sources of burnout and coping mechanism developed by the healthcare workers.

## 2. Methodology

### 2.1. Description of the Study Location

The study was conducted in the Greater Accra Region of Ghana, which lies on the southeastern part of the country. The region occupies a total land area of 3,245 sq. km. It is the national capital of the 16 political regions in Ghana. It has a population density of 1,235.8 people per sq. km. The region is 90.5% urban with an annual urban growth rate of 3.1%. It experiences more inflows of people from other parts of the country than people moving out from the region [14].

### 2.2. Study Design and Sample Size

The study employed the cross-sectional design to obtain quantitative data via pretested questionnaires. The study was carried out in twelve (12) healthcare facilities in the national capital of Greater Accra Region of Ghana. The study population included health workers in the hospitals who fell under the following categories: doctors, nurses, pharmacists, medical laboratory scientists, and radiographers.

The sample size was determined using Miller and Brewer's mathematical formula for estimating single proportions [15]. The standard normal deviation was set at a 95% confidence level, prevalent with the allowable margin of error of 0.08. The formula *n* = *N*/1 + *N*(*α*)2 was used to determine a sample size for each hospital. The minimum sample size increased and rounded up when 10% of the calculated minimum sample size was added for nonresponse and inappropriately filled or missing questionnaires since the questionnaires were interviewer administered. In the formula, *n* is the sample size, *N* is the total population, and *α* is the margin of error, adopted from Miller and Brewer [15]. Thus, a total of 385 questionnaires were distributed for the study. However, 365 were completely filled and returned. This represent a 95% response rate.

### 2.3. Sampling Technique

The study utilized a stratified sampling technique to obtain the required number of respondents from each of the five (5) category of healthcare workers. Thus, in selecting the respondents, sampling proportionate to size was used to determine the number of healthcare workers to be interviewed from each category of healthcare workers.

### 2.4. Data Collection and Analysis

This study took place between September 2018 and December 2018. A standardized questionnaire developed by Pines et al. was used [16, 17] to obtain data. Field inspection of questionnaire data was carried out days after the interview was conducted, and any errors were immediately verified and corrected. The survey instrument comprised 21 questions to be answered on a seven-point Likert scale. Burnout scores were calculated as previously reported [16] and categorized into four levels: very good to good (less than 3), alarming (3 to 3.9), burnout (4 to 5), and acute crisis (more than 5). Furthermore, the questionnaire also captured demographic data of the respondents. It took approximately 25–35 minutes to complete the instrument. Six experts in social science measurement and evaluation determined face validity of the instrument. The average overall face validity was equal to 95%. The study used Cronbach's alpha test formula to test the reliability of the standard questionnaire (Pines et al. burnout questionnaire). The test yielded a reliability coefficient of 0.8. Cronbach's alpha test assesses the internal consistency of a set of scale or of items to ensure that they are all consistent in measuring the same attributes under study [14].

### 2.5. Ethical Considerations

The protocol for the study was ethical and was cleared by the Ethics Review Committee of the Ghana Institute of Management and Public Administration. Prior to data collection, respondents' written and verbal consent was sought. Respondents were informed about the purpose of the study and were made to understand that participation was voluntary and refusal to participate in the study would not affect their employment status. The study respondents were assured of confidentiality and informed that they could withdraw from the study at any time and were at liberty not to answer any question they did not want. All respondents were advised that completing the survey implied informed consent to use the data for research purposes. In addition, all personal identifiers were removed in the summary data to ensure confidentiality.

### 2.6. Data Handling and Analysis

The data were entered into a spreadsheet and later exported to SPSS version 23 and coded for analysis. The analysis included both descriptive and inferential statistics.

Descriptive statistics (frequencies, means, and standard deviations) were used to describe the variables of interest. Univariate analysis was used in obtaining the frequency of sociodemographic characteristics and other discrete variables of the study population. Data were analyzed by contingency tables except for *t*-tests as appropriate for continuous data (for example, age). The chi-squared (*χ*^2^) tests were used to assess the bivariate relationships between these factors as well as for difference in proportions and for other categorical variables. Other descriptive statistics such as the absolute and relative frequency, arithmetic mean, standard deviation (SD), and median (MED) were also computed [18, 19].

All statistical tests were two-tailed, and alpha = 0.05 or less was considered statistically significant.

## 3. Results

### 3.1. Sociodemographic Characteristics

The sociodemographic characteristics of respondents are presented in Table 1. The research revealed that more than half of the respondents (52.1%) were between the ages of 20 and 30 years. While 207 (56.7%) of them were females, most of them were Christians (93.7%) and were single (55.34%). Most of the respondents were Akans (56.7%), and most of them (41.4%) had bachelor's degree as their educational qualification. It was also observed that most of the respondents were nurses (65.2%), and 49.3% of them have 1-5 years of working experience. Moreover, 54.0% of the respondents work in district hospitals, with 58.3% working in the outpatient department.

Table 2 represents the burnout scores among various health worker groups: doctors, nurses, pharmacists, laboratory scientists, and radiographers. The total score for all burnout variables among health worker groups ranged from good (71.50%), alarming (12.60%), acute crisis (6.02%), and burnout (9.90%). Among the health worker groups (doctors, nurses, pharmacists, laboratory scientists, and radiographers), nurses had the highest percentage score values for all burnout variables: good, alarming, acute crisis, and burnout (48.77%, 6.85%, 2.74%, and 6.90%, respectively), followed by doctors and the least representing radiographers. Radiographers reported the same least score value (0.27%) for alarming, acute crisis, and burnout score. Acute crisis and burnout scores were reported lowest for both pharmacists and radiographers (0.27%, respectively). Laboratory scientists indicated the same percentage score value of 0.55% for alarming and acute crisis scores.

Table 3 shows differences in the degree of burnout and some selected sociodemographic characteristics. Among the health worker groups, female respondents reported the highest burnout (63.89%) as against their male counterparts (36.11%). Respondents who were married had higher rates (58.33%), followed by those who were single (27.78%) and the least representing widow/widower (5.56%). Within the educational background of the health worker groups, respondents who had a bachelor's degree exhibited higher (30.56%) burnout, followed by those with diploma qualification (25.00%). The respondents with the least burnout score among the education category were those with PhD qualification (2.78%). Health workers stationed at the inpatient departments showed higher burnout levels (63.89%) as against those who worked at the outpatient departments (36.11%). Health workers who have children (parenthood) have higher burnout rate (58.33%) as against those who did not have any children (41.67%).

Table 4 presents the results of influence of sociodemographic characteristics on burnout among the health workers. The results show that there was an association between burnout and sociodemographic qualities: age (*p* < 0.001), gender (*p* = 0.003), educational qualification (*p* < 0.001), occupation (*p* < 0.001), years of work experience (p < 0.001), marital status (*p* < 0.001), and parenthood (having children) (*p* < 0.001).

Table 5 presents the correlation matrix of the relationship between selected demographic characteristics and burnout. Among the positive correlations, the following variables were significantly different (*p* < 0.05) between each other: age and marital status (0.054) and occupation and children (0.039).

Table 6 shows the multiple logistic regression model for the influence of sociodemographic characteristics on burnout. The results show that females were 1.2 times more likely than males to experience burnout. Respondents between the ages of 41 and 50 were more likely to experience burnout when working than those in the other age groups. There was however a more significant and appreciable relationship between burnout and age: 41-50 years (*p* = 0.041). Regarding educational qualification, the results indicate burnout increases with level of education. Also, a significant relationship was established between nurses and burnout: *p* = 0.050. The results also show that those who had worked at the hospital for 6-10 years were 3.7 times more likely to experience burnout.

**Figure 1:**
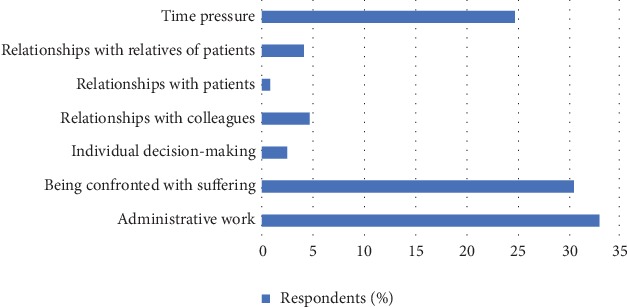
Sources of burnout.

Figure 1 shows the sources of perceived burnout among the health workers. Result shows that most of the health workers' experience indicated that burnout was from administrative work (32.88%), followed by being confronted with suffering (30.41%) and time pressure (24.66%) in that order. The least pressure encountered was from relationships with patients (0.82%) followed by individual decision-making (2.47%). Burnout from relationships with colleagues and relatives of patients (4.66% and 4.11%, respectively) was barely rare.

**Figure 2:**
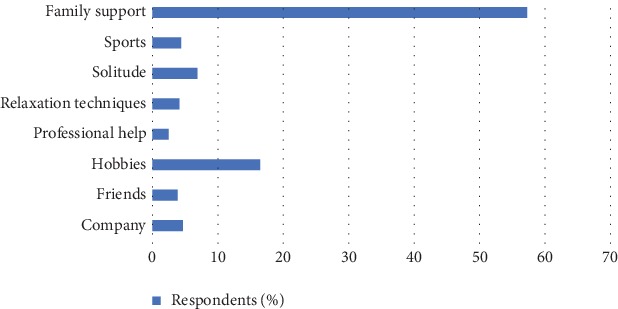
Most common defense against burnout.

The most common defense against burnout is represented in Figure 2. Most of the health workers reported that they are able to get support from family (57.26%) to minimize burnout. This is followed by those who use their interest/hobbies (16.44%) to minimize the effect of burnout. The least defense against burnout was professional help (2.47%). Other defenses against burnout include company (4.66%), friends (3.83%), relaxation techniques (4.11%), solitude (6.85%), and sports (6.85%).

## 4. Discussions

The objective of this study was to determine the levels of burnout experienced by healthcare workers, aimed at identifying the various sources of burnout and coping mechanism developed by the healthcare workers. The assessment was done among 4 different groups of health workers (nurses, doctors, pharmacists, laboratory scientists, and radiographers). Just as it is in most occupations, health workers also go through some form of tiredness or exhaustion. However, persistent frustrations and thwarts on the work of people could turn exhaustion to burnout [20]. The extent of burnout among the various groups of health professionals was found to be 9.90%. This confirms reports indicating the presence of burnout among various healthcare providers in low- and middle-income countries [21]. On the other hand, results from this current study disagree with a similar study by Pavelková and Bužgová [22], Whitebird et al. [23], and Alkema et al. [24] where burnout scores were low.

The challenges and nature of healthcare affect the psyche of these healthcare workers. Thus, in addition to addressing psychological factors, management of hospitals should improve physical working conditions of health workers and engage them in various exercises occasionally to prevent burnout situations [6, 21, 25].

There was an association between burnout and sociodemographic qualities: gender, age, educational qualification, occupation, years of experience, marital status, and children. Several similar studies have showed female health workers having more vulnerability to emotional exhaustion than males and as a result were more prone to burnouts than their male counterparts [26, 27]. Similar explanations could be attributed to what was reported in this study where females had 1.2 times more vulnerability to burnouts than males (Table 6).

From the current study, it was revealed that among the occupations in health institutions, nurses had the highest vulnerability in experiencing burnouts. Chou et al. [28] in their study on job burnout and burnout in hospital employees identified nurses as the most burnt-out among health workers in hospitals at Taiwan. This is understandable because nurses deal with deaths daily, go through emotional challenges of losing patients regularly, are constantly faced with consoling grieving relatives of patients, and sometimes have to go on relatively long shifts thereby causing emotional exhaustion or burnouts [29–31]. Generally, it is widely known that females dominate the nursing occupation [32]. The higher number of females in the nursing fields as compared with males coupled with a higher burnout partly explains the strong correlation between occupation and gender (Table 5).

It was also found from this current study (Table 6) that older people between 41 and 50 years were more vulnerable to burnouts than the other age groups. This is in agreement with the work done by Bijari and Abassi [33], where they found that that older health workers aged 40-50 have a greater subjection to psychological and physical oppressions caused by fatigue resulting from overworking and carrying out tedious duties.

Irrespective of the health worker group, it was revealed from this study that the higher the working experience, the more likely it is for any health professional to encounter burnouts. Work experience may not have a direct influence on burnouts but could be a mediator of the other sociodemographic factors. Furthermore, it was revealed that health workers who are parents or married tend to suffer burnouts more than those who are single. Explanations for this observation include the extra responsibilities, frustrations, and sometimes emotional challenges encountered by parents or married health workers.

The causes of burnout identified by the respondents were administrative work, being confronted with suffering, individual decision-making, relationship with colleagues, relationship with patients, relationship with relatives of patients, and time pressure. However, the main causes were administrative work (32.88%), being confronted with suffering (30.41%), and time pressure (24.66). Pavelková and Bužgová [22] in their study on burnout among hospital workers in Czech Republic also indicated administrative work, being confronted with suffering, and time pressure as the main causes of burnout. Also, their study and this one identified relationship with patients as the least distressing. Workers may consider administrative work pointless and distressing if supervisors or superiors show incompetency, do not give feedbacks, and also make irrelevant changes in hospital regulations [2, 22].

The current studies showed health workers prevented burnout mainly by support from family members and interests/hobbies which was consistent with a similar study by Pavelková and Bužgová [22] and Funk [8]. This observation stands to reason because families are sources of all kinds of support including moral, emotional, financial, and physical support. Hobbies on the other hand may provide emotional upliftment but may not provide all the other forms of support that the family can render. However, it is worth stating that in a study done by White bird et al. [23] on hospital workers in Minnesota, they found out that workers mainly used physical activities as a stress reliever. It is therefore important that each health worker identifies an activity that will best help reduce the work burnout they encounter.

## 5. Study Limitations

In this study, levels of burnout experienced by healthcare workers were assessed; the various sources of burnout and coping mechanism developed by the healthcare workers were also identified, using a standardized questionnaire. First, the results from the cross-sectional study only refer to one point of time. It was performed in only twelve (12) healthcare facilities, thus limiting the generalization of the results found. Another important factor to consider is that although the respondents answered self-administered questionnaires based on their actual performance, overestimation or exaggeration may exist as a questionable factor.

## 6. Conclusion

Though the current study reported burnout among professional health workers in the Greater Accra Region of Ghana, there was an association between burnout and sociodemographic qualities. The main sources of burnout by the workers were administrative work, confrontation with sufferings, and pressure due to time. Based on the findings of these, it is recommended that measures should be put in place in hospitals to assess burnout and burnout levels to ensure people who are going through such situations are properly cared and supported for. Finally, the duties and responsibilities given to nurses and aged workers should be revised regularly.

## Figures and Tables

**Table 1:** Sociodemographic characteristics of respondents.

Variable (*n* = 365)		Frequency	Percentage (%)
Age of respondent			
≤19	3		0.9
20-30	190		52.1
31-40	93		25.6
41-50	54		14.4
51-60	25		6.9
Gender			
Male	158		43.3
Female	207		56.7
Religion			
Christianity	342		93.7
Islam	18		4.9
Traditional	3		0.8
Other	2		0.5
Ethnicity			
Akan	207		56.7
Ga-Dangme	73		20.0
Mole-Dagbon	18		4.9
Ewe	58		15.9
Others	9		2.5
Marital status			
Single	202		55.34
Married	151		41.37
Divorced	3		0.82
Separated	1		0.27
Widow/widower	8		2.19
Educational qualification			
Certificate	92		25.2
Diploma	91		24.9
Bachelor's degree	151		41.4
Master's degree	25		6.8
Others	5		1.4
Occupation			
Doctor	67		18.4
Nurse	238		65.2
Pharmacist	30		8.2
Biomedical	23		6.3
Radiographer	7		1.9
Years of work experience			
1-5 years	180		49.3
6-10 years	98		26.8
11-15 years	41		11.2
16-20 years	19		5.2
21-25 years	16		4.4
>25 years	11		3.0
Category of hospital			
Teaching hospital	20		5.5
Regional hospital	75		20.5
District hospital	197		54.0
Polyclinic	71		19.5
Others	2		0.5
Department of respondents			
Inpatients	152		41.6
Outpatients	213		58.3
Children			
Yes	231		63.29
No	134		36.71

Others under the religion column refers to minority religious bodies such as Hindus. In case of ethnicity, it includes minority ethnic groups. Others under the category of hospitals refers to quasigovernment hospitals.

**Table 2:** Burnout score among the health worker groups.

Burnout score	Doctors (*n* = 67)	Nurses (*n* = 238)	Pharmacists (*n* = 30)	Laboratory scientists (*n* = 23)	Radiographers (*n* = 7)	Total (*n* = 365)	Significance test
	*n* (%)	*n* (%)	*n* (%)	*n* (%)	*n* (%)	*n* (%)	
Good	38 (10.41)	178 (48.77)	25 (6.85)	16 (4.38)	4 (1.09)	261 (71.50)	0.789
Alarming	15 (4.11)	25 (6.85)	3 (0.82)	2 (0.55)	1 (0.27)	46 (12.60)	
Acute crisis	8 (2.19)	10 (2.74)	1 (0.27)	2 (0.55)	1 (0.27)	22 (6.02)	
Burnout	6 (1.64)	25 (6.90)	1 (0.27)	3 (0.82)	1 (0.27)	36 (9.90)	

**Table 3:** Differences in the degree of burnout and selected sociodemographic characteristics.

Variable (*n* = 36)	Burnout	MED	*p*
	*N* (%)		
Gender			
Male	13 (36.11)	2.7	0.386
Female	23 (63.89)	2.6	
Marital status			
Single	10 (27.78)	3.1	0.978
Married	21 (58.33)	2.8	
Divorced	2 (5.56)	2.7	
Separated	1 (2.78)	2.7	
Widow/widower	2 (5.56)	2.6	
Educational qualification			
Certificate	6 (16.67)	2.7	0.345
Diploma	9 (25.00)	2.8	
Bachelor's degree	11 (30.56)	3.2	
Master's degree	5 (13.89)	2.7	
PhD	1 (2.78)	2.1	
Others	4 (11.11)	2.6	
Department of respondents			
Inpatients	23 (63.89)	2.7	0.768
Outpatients	13 (36.11)	2.6	
Children			
Yes	21 (58.33)	2.8	0.953
No	15 (41.67)	2.7	

MED = median.

**Table 4:** Chi-squared test of association between burnout and related characteristics.

Background characteristic	Chi-square (*χ*^2^)	*p* value
Gender	3.093	0.003^∗^
Age	6.649	0.001^∗^
Educational qualification	10.186	0.001^∗^
Occupation	12.990	0.001^∗^
Years of work experience	11.784	0.001^∗^
Marital status	15.742	0.001^∗^
Children	19.124	0.001^∗^

^∗^Significant at 0.05.

**Table 5:** Correlation between selected variables and burnout.

Variable	B	G	A	EQ	O	WK	MS	C
Burnout (B)	1	0.582^∗∗^	-0.322	-0.764	0.587	0.873	0.223	0.439
Gender (G)	0.582^∗∗^	1	0.177	-0.762	0.782	0.718	0.222	0.148
Age (A)	-0.322	0.177	1	0.102	0.321	-0.174	0.054	-0.588
Educational qualification (EQ)	-0.764	-0.762	0.102	1	-0.841	-0.819	-0.292	-0.335
Occupation (O)	0.587	0.782	0.321	-0.841	1	0.716	0.358	0.039
Years of work experience (WK)	0.873	0.718	-0.174	-0.819	0.716	1	0.532	0.383
Marital status (MS)	0.223	0.222	0.054	-0.292	0.356	0.532	1	0.098
Children (C)	0.439	-0.148	-0.588	-0.335	0.039	0.383	0.098	1

^∗∗^Correlation is significant at *p* < 0.01 level (2-tailed). ^∗^Correlation is significant at *p* < 0.05 level.

**Table 6:** Multiple logistic regression model for the influence of sociodemographic characteristics on burnout.

Variable	OR	95% CI	*p* value
Sex			
Female	Ref	26.2	
Male	1.273	0.557-2.908	0.567
Age			
≤19	Ref	28.2	
20-30	1.136	0.372-3.989	0.740
31-40	1.266	0.227-4.399	0.331
41-50	1.386	0.158-0.962	0.041^∗^
51-60	0.195	0.147-1.063	0.066
Education			
Others	Ref	27.6	
Certificate	1.088	0.456-2.597	0.850
Diploma	1.136	0.272-3.989	0.631
Bachelor's degree	1.186	0.357-4.209	0.544
Master's degree	1.264	0.237-4.726	0.662
PhD	1.366	0.337-4.899	0.834
Occupation			
Doctor	Ref	36.8	
Nurse	1.426	0.176-1.031	0.050^∗^
Pharmacist	1.138	0.256-4.597	0.344
Biomedical	1.236	0.232-3.589	0.562
Radiographer	1.038	0.256-4.597	0.927
Years of work experience			
1-5 years	Ref	31.6	
6-10 years	3.789	1.043-21.990	0.064
11-15 years	1.386	0.457-4.209	0.566
16-20 years	1.164	0.497-2.726	0.727
21-25 years	1.136	0.272-3.989	0.544
>25 years	1.266	0.327-4.899	0.567
Department of respondents			
Inpatients	Ref	26.7	
Outpatients	1.432	0.044-4.198	0.469
Children			
Yes	Ref	28.4	
No	1.441	0.168-1.152	0.498

^∗^Significant at 0.05. OR = odds ratio; 95% CI = 95% confidence interval; Ref = reference category.

## Data Availability

The data used to support the findings of this study are included within the article.
